# Antiplatelet Therapy in Atherothrombotic Diseases: Similarities and Differences Across Guidelines

**DOI:** 10.3389/fphar.2022.878416

**Published:** 2022-04-27

**Authors:** Georges Jourdi, Guillaume Marquis-Gravel, Anne-Céline Martin, Marie Lordkipanidzé, Anne Godier, Pascale Gaussem

**Affiliations:** ^1^ Research Center, Montreal Heart Institute, Montreal, QC, Canada; ^2^ Faculty of Pharmacy, Université de Montréal, Montreal, QC, Canada; ^3^ Faculty of Medicine, Université de Montréal, Montreal, QC, Canada; ^4^ Université Paris Cité, INSERM, Innovative Therapies in Haemostasis, Paris, France; ^5^ Service de Cardiologie, AP-HP, Hôpital Européen Georges Pompidou, Paris, France; ^6^ Service d’Anesthésie Réanimation, AP-HP, Hôpital Européen Georges Pompidou, Paris, France; ^7^ Service d’Hématologie Biologique, AP-HP, Hôpital Européen Georges Pompidou, Paris, France

**Keywords:** aspirin, clopidogrel, ticagrelor, prasugrel, atherothrombotic diseases, antiplatelet therapy, acute coronary syndrome, stroke

## Abstract

Antiplatelet therapy, mainly consisting of aspirin and P2Y_12_ receptor antagonists, is the cornerstone of the pharmacological treatment and prevention of atherothrombotic diseases. Its use, especially in secondary cardiovascular prevention, has significantly improved patient clinical outcomes in the last decades. Primary safety endpoint (i.e., bleeding complications) remain a major drawback of antiplatelet drugs. National and international societies have published and regularly updated guidelines for antiplatelet therapy aiming to provide clinicians with practical recommendations for a better handling of these drugs in various clinical settings. Many recommendations find common ground between international guidelines, but certain strategies vary across the countries, particularly with regard to the choice of molecules, dosage, and treatment duration. In this review, we detail and discuss the main antiplatelet therapy indications in the light of the different published guidelines and the significant number of recently published clinical trials and meta-analyses and highlight the areas that deserve further investigation in order to improve antiplatelet therapy in patients with atherothrombotic diseases.

## Introduction

Cardiovascular diseases (CVD) are the most common cause of mortality, responsible for one in 4 deaths globally (Virani et al., 2021). Since platelets play a key role in arterial thrombus formation, antiplatelet agents are one of the most prescribed therapies in medicine. They are mainly indicated for the treatment and prevention of acute coronary syndromes (ACS), stable coronary artery diseases (CAD), peripheral artery diseases (PAD), ischemic stroke and transient ischemic attack (TIA). Although less frequently, they can also be used in patients suffering from other pathologies such as pre-eclampsia, Buerger’s disease and myeloproliferative neoplasms. Clinical practice guidelines for antiplatelet therapy management are published by national and international societies such as the European Society of Cardiology (ESC), the American College of Cardiology (ACC), the American Heart Association (AHA), the Canadian Cardiovascular Society (CCS), the Japanese Circulation Society [JCS, whose guidelines are designed to be used as a reference in Asian countries (Nakamura et al., 2020)], the European Stroke Organization (ESO) and the American Stroke Association (ASA) (non-exhaustive list). They provide physicians with practical recommendations for the best management strategies of patients with given conditions. While CCS recommendations were developed using the Grading of Recommendations, Assessment, Development, and Evaluation (GRADE) approach (Supplementary Table S1) (Mehta et al., 2018), classes of recommendations (COR) and the levels of evidence (LOE) of the other aforementioned guidelines are graded according to the weight of evidence/agreement to indicate, suggest, consider or not recommend a treatment strategy (Supplementary Table S2) and to the sources of available data on a given therapy (single or multiple randomized clinical trials, meta-analyses, non-randomized studies, consensus of experts’ opinions, small studies, registries, and/or retrospectives studies) (Supplementary Table S3). Antiplatelet treatment strategies vary across the guidelines, particularly with regard to the choice of molecules, dosage, combinations, and treatment duration. In this review, we detail and discuss the recommended antiplatelet strategies in various clinical settings across countries in the light of the significant number of clinical trials and meta-analyses that have been recently published or are still ongoing, and highlight areas that deserve further investigation in order to appropriately manage patients with atherothrombotic diseases.

## Acute Coronary Syndromes

### Choice of Antiplatelet Drugs

**FIGURE 1:**
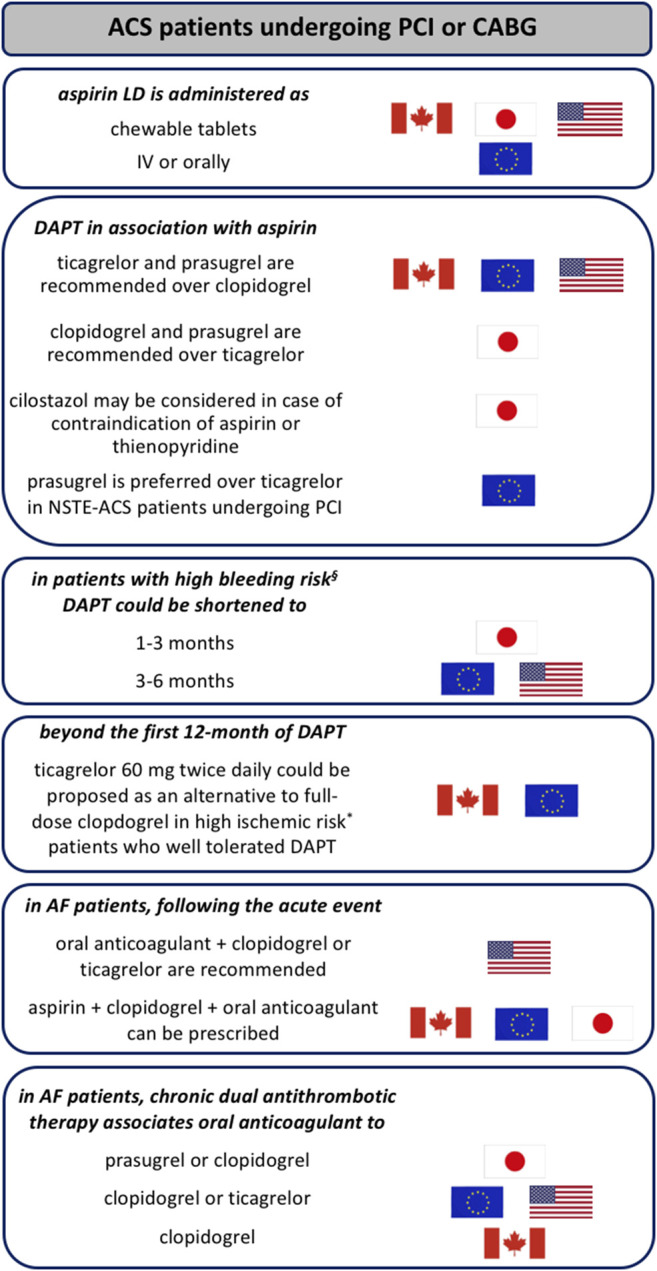
Main differences in antiplatelet therapy recommendations in ACS patients across guidelines. § in case of history of prior bleeding, oral anticoagulant therapy, female sex, advanced age, low body weight, chronic kidney disease, diabetes mellitus, anemia, chronic steroid or nonsteroidal anti-inflammatory drugs (NSAID) therapy (American guidelines); having PREdicting bleeding Complications In patients undergoing Stent implantation and subsEquent Dual Anti Platelet Therapy (PRECISE-DAPT) score ≥25 or meeting the Academic Research Consortium for High Bleeding Risk (ARC-HBR) criteria (European guidelines); having PRECISE-DAPT score ≥25 or DAPT score <2 (given as examples since limited data are available to evaluate the applicability of these scoring systems to the Japanese population; Japanese guidelines). * having at least one clinical (prior myocardial infarction, troponin-positive acute coronary syndrome, diabetes mellitus treated with oral hypoglycemics or insulin, chronic kidney disease with creatinine clearance <60 ml/min, previous stent thrombosis or current smoker) or angiographic features (multiple stents (≥3 stents implanted, ≥3 lesions stented), use of a biodegradable vascular scaffold, long lesion length (>60 mm total stent length), complex lesions (bifurcation treated with 2 stents, stenting of chronic occlusion), left main or proximal left anterior descending artery stenting or multivessel PCI) (Canadian guidelines); having prior stent thrombosis on adequate antiplatelet therapy, stenting of the last remaining patent coronary artery, diffuse multivessel disease especially in diabetic patients, chronic kidney disease (with creatinine clearance <60 ml/min), at least three stents implanted, at least three lesions treated, bifurcation with two stents inplanted, total stent length >60 mm, or treatment of a chronic total occlusion (European guidelines). ACS: acute coronary syndrome; AF: atrial fibrillation; CABG: coronary artery bypass graft; DAPT: dual antiplatelet therapy; LD: loading dose; NSTE: non-ST elevation; PCI: percutaneous coronary intervention.

To prevent major adverse cardiovascular events (MACE) including cardiovascular death, stent thrombosis, myocardial infarction and stroke in ACS patients, dual antiplatelet therapy (DAPT) associating aspirin to a P2Y_12_ receptor antagonist has been the cornerstone of antithrombotic management since the ISAR, STARS and CURE trials (Schömig et al., 1996; Leon et al., 1998; Yusuf et al., 2001). Aspirin has been proven to reduce the relative risk of mortality by 23% after 5 weeks of use and that of myocardial infarction by 25% (Antithrombotic Trialists’, 2002). It is recommended as soon as possible for all patients without contraindications [CCS: strong recommendation, high-quality evidence (Mehta et al., 2018)] at a loading dose (intravenously in Europe or chewable tablets in North America) followed by a daily maintenance dose (Table 1; Figure 1) [ESC: COR I, LOE A (Collet et al., 2021); ACC/AHA: COR I, LOE B-NR (Levine et al., 2016)]. The JCS recommends a first dose of chewable aspirin tablets (162–324 mg) in naïve patients in whom ACS is clinically strongly suspected followed by a loading dose before primary percutaneous coronary intervention (PCI) then by a daily maintenance dose indefinitely unless contraindicated (JCS: COR I, LOE A) (Nakamura et al., 2020). Interestingly, the open-label patient-centered ADAPTABLE trial randomized 15 000 patients with established atherosclerotic CVD (of which 35% had a prior history of myocardial infarction, and 53% a prior coronary revascularization) to either 81 mg or 325 mg of aspirin daily (Marquis-Gravel et al., 2020a). No significant differences in ischemic cardiovascular events or major bleeding between patients of both groups was reported, which further supports the recommendation for 81 mg or 75–100 mg of chronic daily aspirin in clinical practice (Jones et al., 2021).

**TABLE 1:** Recommended doses of antiplatelet agents in ACS patients.

Molecule	Loading dose	Maintenance dose
Aspirin	150–300 mg oral^a^	75–100 mg/day (ESC)
		81 mg (75–100 mg)/day (ACC/AHA)
	75–250 mg IV^b^	81 mg/day (CCS)
		81–162 mg/day (JCS)
Clopidogrel	600 mg oral^c^	75 mg/day
Prasugrel	60 mg oral^d^	10 mg/day
		5 mg/day (if ≤ 60 kg or ≥75 years)^e^
Ticagrelor	180 mg oral	90 mg *b.i.d.*
Cangrelor	30 μg/kg IV	4 μg/kg/min (for at least 2 h or the duration of the procedure)
Eptifibatide	double bolus 180 μg/kg (at 10 min interval)	2 μg/kg/min (for up to 18 h)
Tirofiban	25 μg/kg (over 3 min)	0.15 μg/kg/min (for up to 18 h)

a According to ACC/AHA, and ESC, guidelines; 162–325 mg according to JCS, guidelines.

b According to ESC, guidelines.

c 300 mg oral in patients not receiving reperfusion therapy or in Japanese patients.

d 20 mg oral in Japanese patients.

e 3.75 mg/day in Japanese patients.

ACC, american college of cardiology; AHA, american heart association; CCS, canadian cardiovascular society; ESC, european society of cardiology; IV, intravenous; JCS, japanese circulation society.

Prasugrel and ticagrelor are recommended as more potent P2Y_12_ receptor antagonists over clopidogrel since TRITON-TIMI 38 and PLATO trials (Wiviott et al., 2007; Wallentin et al., 2009), in United States (ACC/AHA: COR IIa, LOE B-R), Canada (CCS: strong recommendation, high-quality evidence) and Europe (ESC:COR I, LOE A) for ACS patients [non-ST elevation (NSTE)-ACS or ST elevation myocardial infarction (STEMI)] undergoing PCI or coronary artery bypass graft (CABG) (Mehta et al., 2018; Collet et al., 2021; Levine et al., 2016). On the contrary, Japanese guidelines recommend either clopidogrel or prasugrel in association to aspirin in ACS patients (JCS: COR I, LOE A) (Kimura et al., 2019) and ticagrelor should be indicated only when a patient is intolerant to both of them (JCS: COR IIa, LOE B) (Figure 1) (Nakamura et al., 2020). Moreover, cilostazol may be considered in patients with contraindication to aspirin or thienopyridine antiplatelet drugs according to the JCS guidelines (JCS: COR IIb, LOE C) (Kimura et al., 2019). That said, clopidogrel is still considered in all the guidelines, particularly when prasugrel or ticagrelor are not available, cannot be tolerated, or are contraindicated. While ticagrelor is recommended irrespective of the planned treatment strategy (invasive or conservative) in ACS patients having non-high bleeding risk (ESC: COR I, LEO B; ACC/AHA: COR IIa, LOE B-R), prasugrel should be limited to those undergoing PCI after knowledge of coronary anatomy without any history of stroke (either ischemic or hemorrhagic) based on the results from the TRILOGY-ACS and ACCOAST trials (ESC: COR I, LOE B; ACC/AHA: COR IIa, LOE B-R) (Levine et al., 2016; Collet et al., 2021). The TRILOGY-ACS trial did not demonstrate the superiority of prasugrel over clopidogrel in NSTE-ACS patients treated medically (Roe et al., 2012), and the ACCOAST trial did not demonstrate the superiority of prasugrel pre-treatment vs. administration after coronary anatomy is known and decision to perform PCI is made in NSTE-ACS patients (Montalescot et al., 2013). In addition, no difference was observed in the efficacy and safety profiles between ticagrelor and prasugrel in ACS patients treated with PCI in the PRAGUE-18 randomized trial (Motovska et al., 2016), although this study was stopped prematurely before the intended sample size could be reached. In the open-label ISAR-REACT 5 trial, the incidence of death, myocardial infarction or stroke was significantly lower among patients who received prasugrel compared with ticagrelor, and the incidence of major bleeding was not significantly different between the two groups of ACS patients (Schüpke et al., 2019). This study led to a preference for prasugrel over ticagrelor in NSTE-ACS patients who proceed to PCI in the 2020 ESC guidelines (ESC: COR IIa, LOE B) (Collet et al., 2021). The open-label POPular AGE trial recently suggested that in patients aged 70 years or older presenting with NSTE-ACS, clopidogrel is a favorable alternative to ticagrelor or prasugrel, because it leads to fewer bleeding events without an increase in the combined endpoint of all-cause death, myocardial infarction, stroke, or bleeding (Gimbel et al., 2020). Regulatory trials of ticagrelor and prasugrel excluded STEMI patients who underwent thrombolysis making clopidogrel (300 mg loading dose in patients aged ≤75, followed by 75 mg/day) the P2Y_12_ receptor antagonist commonly used in such patients (ESC: COR I, LOE A; ACC/AHA: COR I, LOE A). However, the multicenter randomized open-label TREAT trial recently evaluated the efficacy of ticagrelor when compared with clopidogrel in STEMI patients (aged <75 years) who have previously received fibrinolytic therapy and showed similar frequency of cardiovascular events (including cardiovascular mortality, myocardial infarction, stroke, severe recurrent ischemia, TIA, or other arterial thrombotic events) and bleeding complications (encompassing major, fatal, and intracranial bleeding) between both groups at 12 months (Berwanger et al., 2019), but guidelines have not yet incorporated the results of this study in their recommendations.

Cangrelor may be an interesting therapeutic option in ACS patients who need urgent PCI and are not pre-treated with an oral P2Y_12_ receptor antagonist (ESC: COR IIb, LOE A) to reduce peri-procedural thrombotic events (Bhatt et al., 2013; Ibanez et al., 2018), however its use in clinical practice remains limited. In the double-blind, placebo-controlled CHAMPION PHOENIX trial, cangrelor significantly reduced the rate of ischemic events, including stent thrombosis in comparison to clopidogrel (given at 300 or 600 mg) with no significant increase in severe bleeding (Bhatt et al., 2013). Many ongoing clinical trials will provide further insights into the comparative role of cangrelor and oral P2Y_12_ receptor antagonists (particularly ticagrelor and prasugrel) in the context of ACS patients. Cangrelor could also be an interesting perioperative bridging strategy in high thrombotic risk patients (typically stent implanted within the last 4 weeks) who need urgent high bleeding risk surgery (Bhattad et al., 2019).

The use of GPIIbIIIa inhibitors in ACS setting has decreased in the last years due to bleeding concerns and the introduction of potent oral P2Y_12_ receptor antagonists. According to the ESC guidelines, circumstances where GPIIbIIIa inhibitors should still be considered in contemporary practice include as a bailout therapy in the event of angiographic evidence of a large thrombus, slow- or no-reflow, and other thrombotic complications in STEMI patients undergoing PCI (ESC: COR IIa, LOE C), although this strategy has not been tested in a randomized trial (Ibanez et al., 2018). They could be considered in case of thrombotic complications in NSTE-ACS patients without pre-treatment with oral P2Y_12_ receptor antagonists however they are not recommended in patients in whom coronary anatomy is not known (ESC: COR III, LOE A) (Neumann et al., 2019; Collet et al., 2021). No compelling evidence for additional benefit of routine upstream use of GPIIbIIIa inhibitors exists in NSTE-ACS patients scheduled for coronary angiography (Collet et al., 2021).

### Antiplatelet Therapy Duration

**FIGURE 2:**
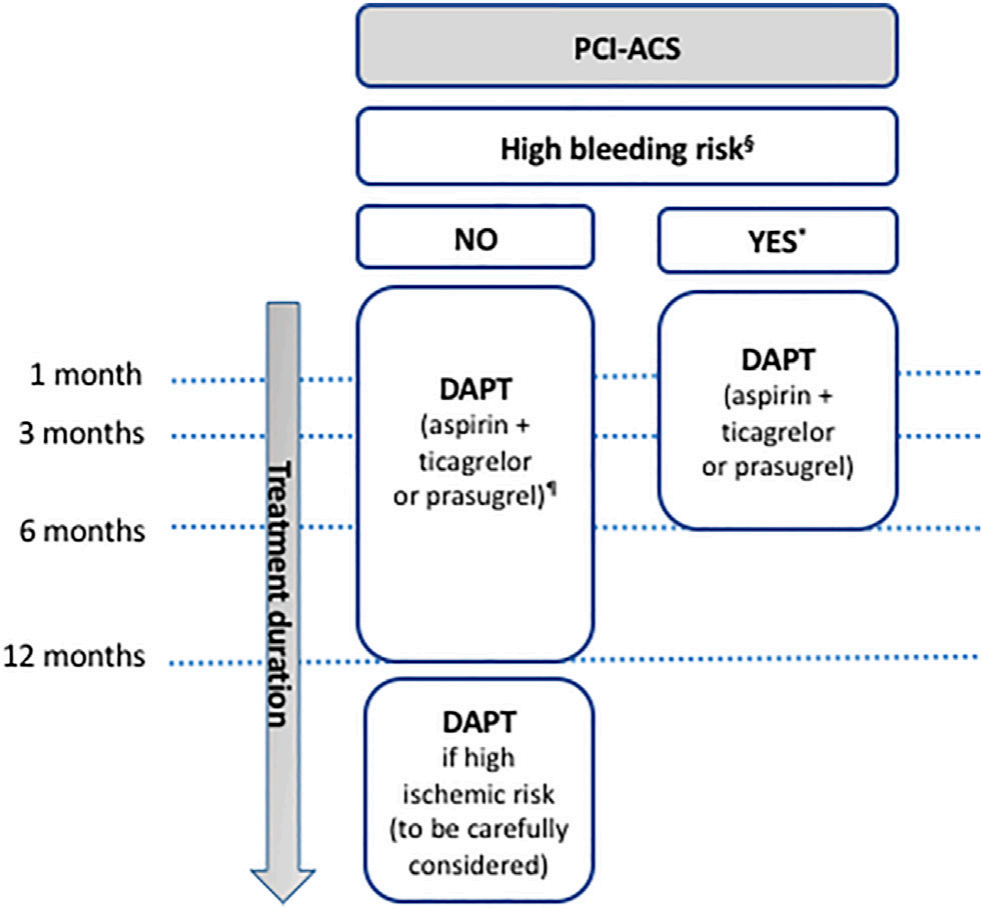
Antiplatelet therapy in ACS patients undergoing PCI. § in case of history of prior bleeding, oral anticoagulant therapy, female sex, advanced age, low body weight, chronic kidney disease, diabetes mellitus, anemia, chronic steroid or nonsteroidal anti-inflammatory drugs (NSAID) therapy (American guidelines); in case of need for oral anticoagulant drugs in addition to DAPT, advanced age (older than 75 years), frailty, anemia with hemoglobin <110 g/L, chronic renal failure (creatinine clearance <40 ml/min), low body weight (<60 kg), hospitalization for bleeding within past year, previous stroke/intracranial bleed or regular need for NSAIDs or prednisone (Canadian guidelines); having PREdicting bleeding Complications In patients undergoing Stent implantation and subsEquent Dual Anti Platelet Therapy (PRECISE-DAPT) score ≥25 or meeting the Academic Research Consortium for High Bleeding Risk (ARC-HBR) criteria (European guidelines); having PRECISE-DAPT score ≥25 or DAPT score <2 (given as examples since limited data are available to evaluate the applicability of these scoring systems to the Japanese population; Japanese guidelines). * except for the Canadian guidelines that do not propose to shorten duration of DAPT in patients at high bleeding risk. ¶ except for the Japanese guidelines that recommend aspirin + clopidogrel or prasugrel. DAPT: dual antiplatelet therapy; PCI: percutaneous coronary intervention; ACS: acute coronary syndrome.

DAPT for 12 months is the default option for all ACS patients (ACC/AHA: COR I, LOE B-R; ESC: COR I, LOE A; CCS: strong recommendation, high-quality evidence; JSC: COR I, LOE A). In high bleeding risk patients, DAPT can be shortened to 1–3 (JSC: COR I, LOE A) or to 6 months (ESC: COR IIa, LOE B (ACS-PCI)/LOE C (ACS-medically managed); ACC/AHA: COR IIb, LOE C-LD) (Levine et al., 2016; Kimura et al., 2019; Valgimigli et al., 2018) (Figure 2) while the CCS does not propose to shorten duration of DAPT in patients at high bleeding risk (Figure 1) (Mehta et al., 2018). Similar type and duration of DAPT are recommended in male and female patients (ESC: COR I, LOE A) (Valgimigli et al., 2018). Moreover, European guidelines specified that DAPT should be considered for at least 12 months in case of ACS patients undergoing PCI with a bioresorbable vascular scaffold (ESC: COR IIa, LOE C) (Valgimigli et al., 2018). The default duration of 12 months DAPT in ACS patients undergoing PCI is based on the CURE trial (Yusuf et al., 2001) although patients have received DAPT for 3–12 months with a mean duration of 9 months in this study. In selected patients at high risk for ischemic events who have tolerated DAPT without bleeding complications, a prolonged DAPT over 12 months [ESC: COR IIb, LOE A (ACS-PCI)/LOE B (ACS-medically managed)/LOE C (ACS-CABG); CCS: strong recommendation, high-quality evidence; ACC/AHA: COR IIb, LOE A; JSC: COR IIb, LOE B] (Mehta et al., 2018; Collet et al., 2021; Levine et al., 2016; Kimura et al., 2019) should be carefully considered due to the high incidence of myocardial infarction, cardiovascular death and ischemic stroke at 1 year after ACS, estimated around 11.4% (Yusuf et al., 2001), which is related to ongoing platelet activation long after clinical stabilization (Ault et al., 1999) as for instance in diabetes mellitus (DM) patients (Knuuti et al., 2020). Indeed, the DAPT trial evaluated the benefits and risk of prolonging DAPT (associating aspirin with a thienopyridine, clopidogrel in most cases) for another 18 months following the first-year after coronary drug-eluting stent (DES) placement. DAPT significantly reduced the risks of stent thrombosis, MACE and cerebrovascular events as compared with aspirin therapy alone at 30 months of treatment at the expense of an increased rate of moderate or severe bleeding. Importantly, the risk of stent thrombosis and myocardial infarction was comparable between both groups during the 3 months after discontinuation of thienopyridine treatment (Mauri et al., 2014). The reduction of ischemic events with DAPT is greatest in the first few weeks due to protection from early stent thrombosis. Over time, the benefit from protection against stent thrombosis decreases, and the predominant advantage of DAPT shifts to protection from spontaneous myocardial infarction, i.e., from plaque rupture at sites remote from the stented index lesion (Mauri et al., 2014). On the other hand, bleeding risk from DAPT is directly proportional to the length of therapy, with longer periods leading to increased bleeding events and higher rates of non-cardiovascular mortality (Palmerini et al., 2015). Indeed, bleeding while on DAPT in ACS patients treated with or without PCI is associated with a similar increase in subsequent all-cause mortality and has an equivalent prognostic impact as myocardial infarction as was recently shown in a pooled dataset analysis of 4 multicenter randomized trials (APPRAISE-2, PLATO, TRACER and TRILOGY ACS) (Marquis-Gravel et al., 2020b) highlighting the importance of bleeding avoidance in parallel to the ischemic protection in order to improve ACS patients prognosis. In light of the PEGASUS-TIMI 54 trial results (Bonaca et al., 2015), regulatory authorities in Canada (CCS: strong recommendation, high-quality evidence) and Europe (ESC: COR IIb, LOE B) approved 60-mg ticagrelor twice daily in association to aspirin as a prolonged DAPT in patients with myocardial infarction and high ischemic risk who have tolerated DAPT without bleeding complication during the first 12 months of treatment (Mehta et al., 2018; Collet et al., 2021) (Figure 1). The international observational study EPICOR demonstrated that 57% of ACS patients were receiving DAPT beyond 12 months (Bueno et al., 2017) and in another registry of patients from the United States and 4 European countries, 43% of ACS patients and 57% of those who underwent elective PCI were receiving DAPT at the end of 2 years of follow-up (Mehran et al., 2013). Upon the completion of DAPT, a chronic daily dose of aspirin is recommended by all guidelines unless contraindicated in which case clopidogrel should be considered.

### De-escalation of Dual Antiplatelet Therapy in Acute Coronary Syndromes Patients

De-escalation of DAPT may be an option in patients with high bleeding risk or those who have experienced clinically significant bleeding complications under antiplatelet therapy, particularly when using potent P2Y_12_ receptor antagonists. In TWILIGHT trial (Mehran et al., 2019), 7,119 patients at high risk for bleeding or ischemic event who had undergone PCI (with ACS as the indication in 64.8% of cases) and had not had a major bleeding or ischemic events during the first 3 months of DAPT (ticagrelor plus aspirin), continued to take ticagrelor and were randomly assigned to receive aspirin or placebo for an additional 12 months. Bleeding rates were significantly lower in ticagrelor monotherapy group compared to continued DAPT group. In addition, ticagrelor monotherapy after 3 months was non-inferior to continued DAPT in terms of ischemic events. Similarly, among patients undergoing PCI with DES (not exclusively ACS patients), P2Y_12_ receptor antagonist monotherapy after 1 (STOPDAPT-2 & GLOBAL LEADERS trials) or 3 months (TICO & SMART CHOICE trials) of DAPT compared with prolonged DAPT (up to 12 months) resulted in non-inferior rates of ischemic events, and a lower (STOPDAPT-2, TICO & SMART CHOICE trials) or similar (GLOBAL LEADERS trial) rate of bleeding complications (Vranckx et al., 2018; Hahn et al., 2019; Watanabe et al., 2019; Kim et al., 2020a). However, data analysis from ACS subgroup of STOPDAPT-2 trial (Watanabe et al., 2019) showed that 1-month DAPT followed by clopidogrel monotherapy for 11 months did not meet criteria for non-inferiority compared with standard 12-months DAPT for the composite of net clinical benefits (a composite of ischemic/bleeding endpoints). Recently, MASTERDAPT trial was the first published study to have compared two short DAPT duration in patients at high risk for bleeding after DES implantation. It revealed that 1 month of DAPT was non-inferior to the continuation of therapy for at least 2 additional months with regard to the occurrence of net adverse clinical events (a composite of death from any cause, myocardial infarction, stroke, or major bleeding) and was associated with a lower incidence of major or clinically relevant non-major bleeding (Valgimigli et al., 2021). In the light of some major differences in the aforementioned trials with respect to early phase design, timing of randomization, timing of aspirin interruption and blinding in the administration of treatments in addition to non-inferiority design and large bleeding definitions, the efficacy and safety of aspirin-free strategies following a short-duration DAPT as compared to 12-months DAPT strategy as well as the optimal timing for de-escalation of DAPT remain an area for future research, especially in terms of targeting the most appropriate population as outlined in the recently published meta-analysis of Giacoppo et al. (Giacoppo et al., 2021).

The efficacy and safety of the antiplatelet therapy de-escalation by switching from ticagrelor to clopidogrel 30 days following DAPT associating aspirin with ticagrelor in patients with acute myocardial infarction without major ischemic or bleeding events during the acute phase after index PCI with new-generation DES, have been evaluated in the large-scale, multicenter, non-inferiority randomized TALOS-AMI trial (Kim et al., 2021). The de-escalation strategy significantly reduced the risk of net clinical events (including cardiovascular death, myocardial infarction, stroke and hemorrhagic complications) up to 12 months, mainly by reducing the bleeding events (Kim et al., 2021). In the second randomized, open-label, multicenter, non-inferiority HOST-REDUCE-POLYTECH-ACS trial, ACS patients undergoing PCI were randomly assigned following 1 month of DAPT combining prasugrel and aspirin to a de-escalation group receiving 5 mg prasugrel or conventional group receiving 10 mg prasugrel in combination to aspirin for up to 12 months. The risk of adverse clinical events (encompassing all-cause death, non-fatal myocardial infarction, stent thrombosis, repeat revascularization, stroke, and bleeding events) was significantly lower in the former group, mainly due to the reduction in bleeding without an increase in ischemia (Kim et al., 2020b). Consequently, de-escalation of DAPT in patients undergoing PCI might be a feasible alternative strategy during chronic maintenance therapy, especially when the risk of bleeding is a concern. It may be done unguided based on clinical judgment or guided by platelet function testing or CYP2C19 genotyping, depending on patient risk profiles and availability of respective assays as highlighted in the latest European guidelines (ESC: COR IIb, LOE A) (Collet et al., 2021). Taking into account the limited number of published trials, alongside their non-inferiority design and their mixed efficacy/safety endpoints (thus largely underpowered to assess anti-thrombotic protection vs. avoiding minor bleeding), more studies are needed to confirm the long-term clinical benefit of such strategies.

### Antiplatelet Therapy in Acute Coronary Syndromes Patients Receiving Anticoagulant Drugs

Special attention should be given to cases where oral anticoagulant drugs are indicated for stroke and systemic embolism prevention, including patients with mechanical heart valves or atrial fibrillation (AF) (Lip et al., 2010; Pisters et al., 2010). Overall, 20%–30% of such patients have concomitant CVD requiring PCI (Dewilde et al., 2013). Indeed, in the Framingham study, development of AF was associated with a doubling of CVD mortality (Kannel et al., 1982). Identification of appropriate antithrombotic therapy and adaptation of its duration in these patients is therefore mandatory.

**FIGURE 3:**
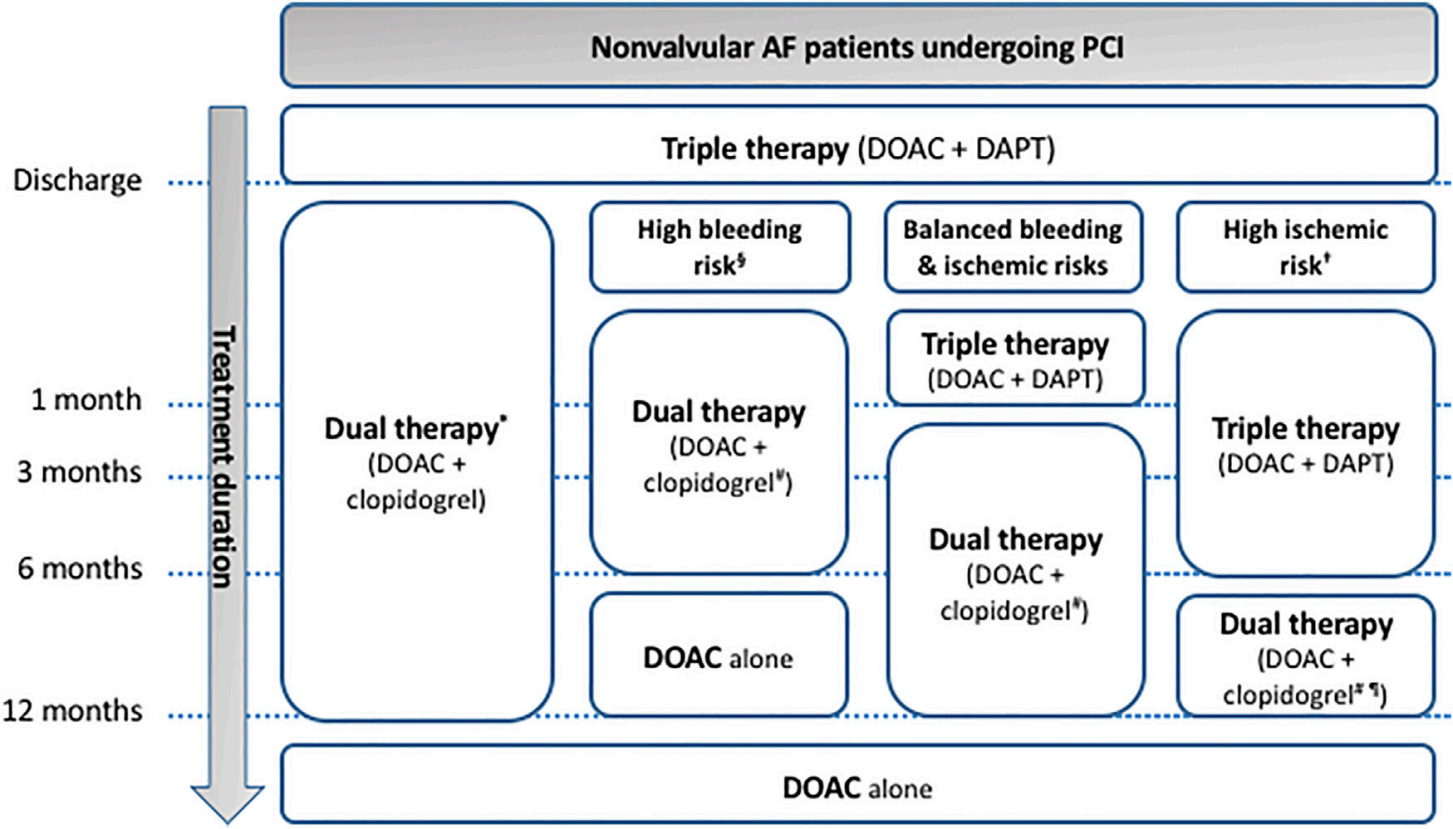
Antithrombotic therapy in nonvalvular AF patients undergoing PCI. * default strategy recommended by the American guidelines irrespective of the bleeding/ischemic risk stratification. § advanced age (older than 75 years), frailty, anemia with hemoglobin <110 g/L, chronic renal failure (creatinine clearance <40 ml/min), low body weight (<60 kg), hospitalization for bleeding within past year, previous stroke/intracranial bleed or regular need for NSAIDs or prednisone (Canadian guidelines); having Hypertension, Abnormal renal and liver function, Stroke, Bleeding history or predisposition, Labile INR, Elderly (>65 years), Drugs and alcohol (HAS-BLED) score ≥3 or Age, Biomarkers (growth differentiation factor-15 (GDF-15), high-sensitivity cardiac troponin T (cTnT-hs) and haemoglobin), and Clinical history (previous bleeding) (ABC) score >2 (European guidelines); having PRECISE-DAPT score ≥25 or DAPT score <2 (given as examples since limited data are available to evaluate the applicability of these scoring systems to the Japanese population; Japanese guidelines). † having at least one clinical (before myocardial infarction, troponin-positive acute coronary syndrome, diabetes mellitus treated with oral hypoglycemics or insulin, chronic kidney disease with creatinine clearance <60 ml/min, previous stent thrombosis or current smoker) or angiographic features (multiple stents (≥3 stents implanted, ≥3 lesions stented), use of a biodegradable vascular scaffold, long lesion length (>60 mm total stent length), complex lesions (bifurcation treated with 2 stents, stenting of chronic occlusion), left main or proximal left anterior descending artery stenting or multivessel PCI) (Canadian guidelines); having prior stent thrombosis on adequate antiplatelet therapy, stenting of the last remaining patent coronary artery, diffuse multivessel disease especially in diabetic patients, chronic kidney disease (with creatinine clearance <60 ml/min), at least three stents implanted, at least three lesions treated, bifurcation with two stents inplanted, total stent length >60 mm, or treatment of a chronic total occlusion (European guidelines); having PRECISE-DAPT score <25 or DAPT score ≥2 (given as examples since limited data are available to evaluate the applicability of these scoring systems to the Japanese population; in Japanese guidelines). # in Japanese guidelines, prasugrel is also allowed in association to DOAC. ¶ in American and European guidelines, ticagrelor might be an alternative to clopidogrel in patients with high ischemic and low bleeding risks. AF: atrial fibrillation; DAPT: dual antiplatelet therapy; DOAC: direct oral anticoagulation; PCI: percutaneous coronary intervention.

Dual therapy associating an oral anticoagulant to a P2Y_12_ receptor antagonist (clopidogrel or ticagrelor) is the default strategy recommended by the AHA/ACC (COR IIa, LOE B-R) after patient discharge following PCI (Kimura et al., 2019; January et al., 2019) while triple therapy combining DAPT (aspirin plus clopidogrel) and oral anticoagulant can be first indicated by ESC (ESC: COR IIa, LOE B), JCS (COR I, LOE C) and CCS (strong recommendation, moderate quality evidence (Nakamura et al., 2020; Mehta et al., 2018; Valgimigli et al., 2018) (Figures 1, 3). The use of ticagrelor or prasugrel is not recommended as part of the triple antithrombotic therapy (ESC: COR III, LOE C). Based on the non-inferior efficacy and the higher (in the PIONEER AF-PCI, RE-DUAL PCI and AUGUSTUS trials) or non-inferior (in the ENTRUST-AF PCI trial) rate of clinically significant bleeding complications associated with the standard triple antithrombotic therapy (aspirin plus P2Y_12_ receptor antagonist plus vitamin K antagonist) in comparison to dual therapy combining direct oral anticoagulant (DOAC) with P2Y_12_ receptor antagonist (Gibson et al., 2016; Cannon et al., 2017; Lopes et al., 2019; Vranckx et al., 2019), duration of the triple therapy is recommended to be as short as possible: during index hospitalization (up to 1 week, when bleeding risk outweighs ischemic risk) (ESC: COR I, LOE A; CCS strong recommendation, moderate-quality evidence; JCS: COR I, LOE C) or up to 1 month (in patients with balanced ischemic/bleeding risks) (ESC: COR IIa, LOE C) or 6 months (in patients with high ischemic risk) for the ESC (COR IIa, LOE B) and CCS (strong recommendation, moderate-quality evidence) (Andrade et al., 2018; Nakamura et al., 2020; Collet et al., 2021) and up to 4–6 weeks for the AHA/ACC (COR IIb, LOE B-R) and JCS (COR III, LOE B) (if triple therapy is selected) (January et al., 2019; Nakamura et al., 2020). It is noteworthy that aspirin was compared with placebo (in addition to the comparison of apixaban vs. vitamin K antagonist) in patients treated with a P2Y_12_ receptor antagonist in the 2 × 2 factorial AUGUSTUS trial. The regimen without aspirin resulted in less bleeding and fewer hospitalizations without significant differences in the incidence of ischemic events than regimen including aspirin (Lopes et al., 2019). However, the risk of coronary ischemic events (including myocardial infarction) was higher (although not significantly) in the placebo group than in the aspirin group. Being underpowered for coronary ischemic event evaluation, interpretation of the AUGUSTUS trial with regard to this issue should be done with caution and large clinical trials evaluating this question are eagerly awaited.

Triple therapy is followed by dual antithrombotic therapy associating DOAC to preferably clopidogrel (ESC: COR I, LOE A; CCS: weak recommendation, low-quality evidence) for 1 year after coronary stenting (ESC: COR I, LOE B; CCS: weak recommendation, moderate-quality evidence) (Andrade et al., 2018; Collet et al., 2021). According to the European consensus document, this dual therapy could be considered as an alternative initial regimen in patients with very high bleeding risk (ESC: COR IIa, LOE A) (Lip et al., 2019). In Japanese guidelines, prasugrel is also allowed in dual therapy because of its low dosage (Saito and Kobayashi, 2020) while in the American and European guidelines, ticagrelor might be an alternative to clopidogrel in patients with a moderate to high ischemic and low bleeding risks irrespective of the type of stent used (ESC: COR IIb, LOE C; ACC/AHA: COR IIa, LOE B-R) (January et al., 2019; Lip et al., 2019; Collet et al., 2021). DOACs are preferred to vitamin K antagonists as oral anticoagulant drugs in AF patients (ESC: COR I, LOE A; CCS: weak recommendation, moderate-quality evidence; ACC/AHA: COR I, LOE A; JCS: COR I, LOE A) (Andrade et al., 2018; January et al., 2019; Knuuti et al., 2020; Nakamura et al., 2020).

For patients with a mechanical heart valves who are managed with a vitamin K antagonist and have an indication for antiplatelet therapy, addition of aspirin 75–100 mg daily may be considered when the risk of bleeding is low according to the American guidelines (ACC/AHA: COR IIb, LOE B-R) (Otto et al., 2021). ESC guidelines recommend a daily dose of clopidogrel in addition to vitamin K antagonist following a one-month triple therapy in patients with mechanical heart valves undergoing PCI (ESC: COR IIa, LOE B) (Baumgartner et al., 2017). Whenever selected, triple therapy could be prolonged up to 6 months (ESC: COR IIa, LOE B; CCS: weak recommendation, very-low-quality evidence) (Baumgartner et al., 2017; Mehta et al., 2018) or even beyond 1 year (JCS: COR IIa, LOE C) (Nakamura et al., 2020) in high ischemic risk patients while the former dual antithrombotic therapy should be considered as an alternative to one-month triple antithrombotic therapy in patients in whom the bleeding risk outweighs the ischemic risk (ESC: COR IIa, LOE A) (Baumgartner et al., 2017). Beyond 1 year of dual therapy, oral anticoagulation should be considered with subsequent withdrawal of antiplatelet agents (ESC: COR IIa, LOE B) (Mehta et al., 2018; Neumann et al., 2019). It should be noted that DOAC are contraindicated in patients with mechanical heart valves (ESC: COR III, LOE A; ACC/AHA: COR III Harm, LOE B-R; CCS: strong recommendation, moderate-quality evidence) (Baumgartner et al., 2017; Mehta et al., 2018; January et al., 2019; Otto et al., 2021).

### Antiplatelet Therapy in Acute Coronary Syndromes Patients Suffering From Diabetes Mellitus

Another population that requires special attention is diabetic patients with ACS. Post hoc analysis of DM patients in the TRITON-TIMI 38 trial showed marked benefit of prasugrel over clopidogrel (Wiviott et al., 2008), while, in PLATO, the absolute benefit of ticagrelor over clopidogrel was greatest in patients with both DM and chronic kidney disease (Franchi et al., 2019). Prasugrel and ticagrelor are therefore preferred over clopidogrel in diabetic ACS patients (ESC: COR I, LOE A) (Cosentino et al., 2020) since DM is associated with increased platelet reactivity seen at baseline and on-treatment (Wiviott et al., 2007; Hamilos et al., 2018). Prolongation of DAPT beyond 12 months with a full-dose clopidogrel or reduced-dose ticagrelor (60 mg b.i.d.) should be considered, for up to 3 years, in DM patients who have tolerated DAPT without major bleeding complications (ESC: COR IIa, LOE A) (Cosentino et al., 2020). Long-term secondary prevention is based on aspirin (ESC: COR I, LOE A) except in patients without high bleeding risk in which the addition of a second antithrombotic drug should be considered (ESC: COR IIa, LOE A) (Cosentino et al., 2020).

## Stable Coronary Artery Diseases

**FIGURE 4:**
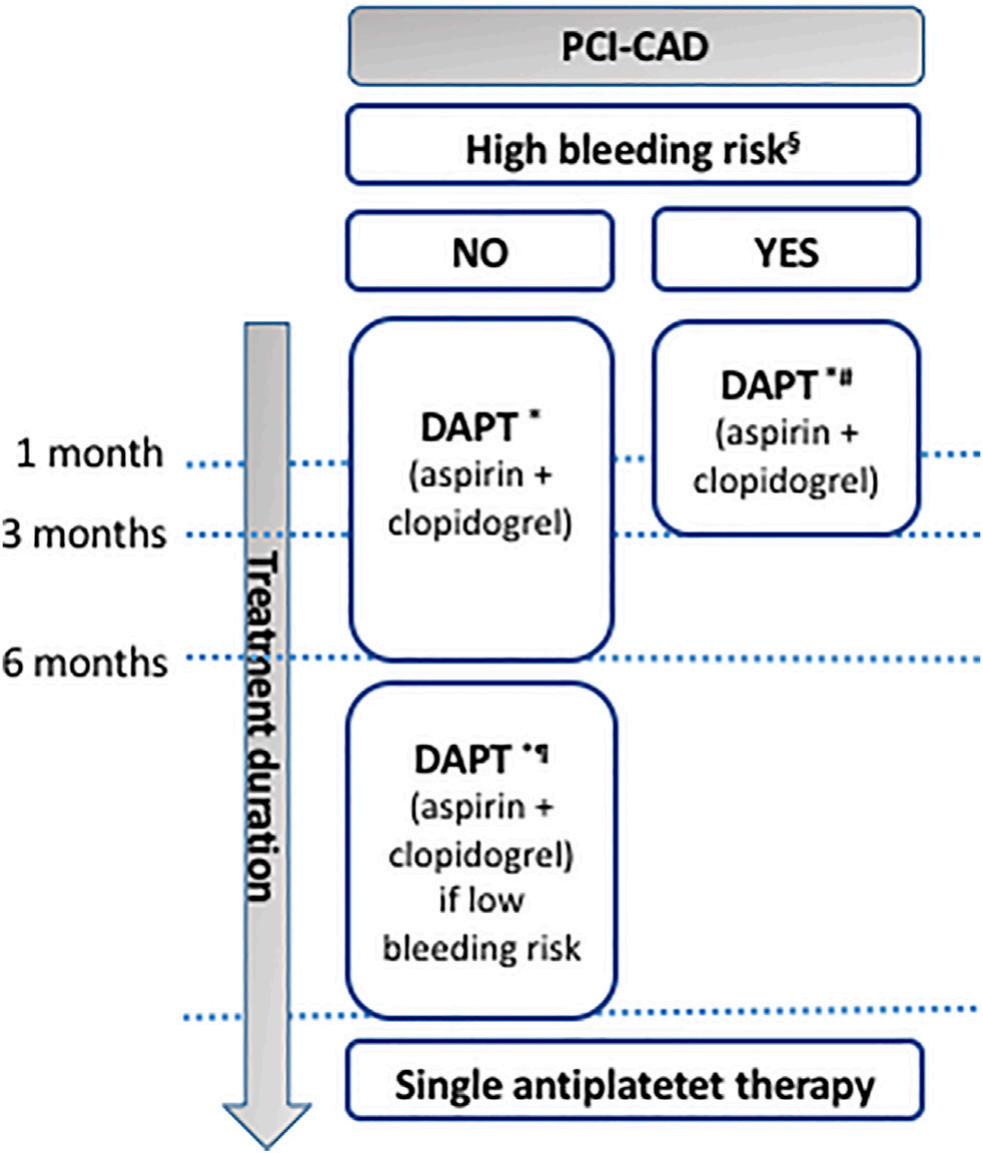
Antiplatelet therapy in CAD patients undergoing PCI. § in case of history of prior bleeding, oral anticoagulant therapy, female sex, advanced age, low body weight, chronic kidney disease, diabetes mellitus, anemia, chronic steroid or nonsteroidal anti-inflammatory drugs (NSAID) therapy (American guidelines); in case of need for oral anticoagulant drugs in addition to DAPT, advanced age (older than 75 years), frailty, anemia with hemoglobin <110 g/L, chronic renal failure (creatinine clearance <40 ml/min), low body weight (<60 kg), hospitalization for bleeding within past year, previous stroke/intracranial bleed or regular need for NSAIDs or prednisone (Canadian guidelines); having PREdicting bleeding Complications In patients undergoing Stent implantation and subsEquent Dual Anti Platelet Therapy (PRECISE-DAPT) score ≥25 (European guidelines); having PRECISE-DAPT score ≥25 or DAPT score <2 (given as examples since limited data are available to evaluate the applicability of these scoring systems to the Japanese population; Japanese guidelines). * Japanese guidelines recommend either clopidogrel or prasugrel. # ESC recommends shortening the duration of DAPT down to 1 month if safety concerns with DAPT are present. ¶ DAPT up to 30 months for the European and Japanese, 36 months for the Canadian, and ≥6 months for the American guidelines. DAPT: dual antiplatelet therapy; PCI: percutaneous coronary intervention.

In current clinical practice, clopidogrel is prescribed in association with aspirin for 6 months in CAD patients undergoing PCI with DES and low to moderate bleeding risk (ESC: COR I, LOE A; CCS: strong recommendation, moderate-quality evidence; ACC/AHA: COR I, LOE B-R) and for 3 months in case of high bleeding risk (ACC/AHA: COR IIb, LOE C-LD; CCS: weak recommendation, low-quality evidence; ESC: COR IIa, LOE B; JCS: COR IIa, LOE B) (Mehta et al., 2018; Levine et al., 2016; Valgimigli et al., 2018) while Japanese guidelines recommend either clopidogrel or prasugrel as the P2Y_12_ antagonist compound in association to aspirin for 1–3 months (JSC: COR I, LOE A) (Nakamura et al., 2020) (Figure 4). ESC recommends shortening the duration of DAPT to 1 month if safety concerns with DAPT are present (ESC: COR IIb, LOE C). On the other hand, DAPT can be extended, particularly in CAD patients with low bleeding risk, for more than 6 months for the ACC/AHA (COR IIb, LOE A), up to 30 months in the ESC (ESC: COR IIb, LOE A) and JCS guidelines (JCS: COR IIb, LOE B) and up to 36 months for the CCS (weak recommendation, moderate-quality evidence) (Levine et al., 2016; Mehta et al., 2018; Valgimigli et al., 2018; Nakamura et al., 2020).

Following DAPT, lifelong single antiplatelet therapy (SAPT) is indicated in CAD patients who have undergone PCI (Levine et al., 2016; Mehta et al., 2018; Valgimigli et al., 2018). Very recently, the efficacy and safety of aspirin or clopidogrel monotherapy during the chronic maintenance period in CAD patients who underwent PCI with DES 6–18 months ago, were compared head to head in the prospective, randomized, open-label, multicenter HOST-EXAM trial (Koo et al., 2021). Clopidogrel was shown to be superior to aspirin in preventing future adverse clinical events encompassing all-cause death, non-fatal myocardial infarction, stroke, readmission due to ACS, or bleeding complications (Koo et al., 2021). However, the primary endpoints of this trial did not include the standard 3 or 4 points MACE but rather a mixture of efficacy and safety endpoints, thus the efficacy/safety superiority of clopidogrel vs. aspirin cannot be clearly ascertained. That said, aspirin remains currently the first choice in this patient population, as it is affordable and widely available even in low-income countries.

COMPASS was the first randomized clinical trial to evaluate the efficacy and safety of rivaroxaban (2.5 mg twice daily) plus aspirin (100 mg once daily), rivaroxaban (5 mg twice daily), or aspirin (100 mg once daily) in the setting of chronic CAD associated or not with PAD. It demonstrated favorable efficacy of dual antithrombotic therapy in terms of thrombotic event reduction, largely driven by a significant reduction in the risk of overall stroke and cardiovascular death, in comparison to aspirin alone, but at the expense of an increased risk of major bleeding (Eikelboom et al., 2017). Consequently, the European Medicines Agency (EMA) and the Food and Drug Administration (FDA) have approved rivaroxaban 2.5 mg twice daily in combination with low-dose aspirin for the prevention of atherothrombotic events in adult patients with CAD and a high risk of ischemic events, while those presenting high bleeding risk like the elderly, those with advanced kidney disease, low body weight, prior bleeding accidents, or a history of stroke or TIA may not be suitable for such therapy. More efforts should be made to accurately define the patient populations that would take real advantage of this strategy, with no unbalanced bleeding risk.

## Peripheral Artery Diseases

In patients with chronic symptomatic PAD, antiplatelet therapy with aspirin alone or clopidogrel alone is recommended to reduce myocardial infarction, stroke, and vascular death (ESC: COR I, LOE A; ACC/AHA: COR I, LOE A) (Gerhard-Herman et al., 2017; Aboyans et al., 2018). The latter may be preferred over the former (ESC: COR IIb, LOE B) (Aboyans et al., 2018). Ticagrelor monotherapy among patients with PAD was assessed in the EUCLID (Hiatt et al., 2017) and SOCRATES (Johnston et al., 2020) studies and showed no significant improvement compared to clopidogrel or aspirin, respectively. In the context of stable PAD patients with polyvascular diseases and prior myocardial infarction, ticagrelor plus aspirin significantly reduced MACE and limb events in comparison to aspirin monotherapy in a sub-study of the PEGASUS-TIMI 54 trial (Bonaca et al., 2016). In the light of the previously mentioned COMPASS trial, the association of rivaroxaban 2.5 mg twice-daily to aspirin 100 mg once daily could be suitable in adult patients with symptomatic PAD at high risk of ischemic events without high bleeding risk as for instance in some DM patients (ESC: COR IIa, LOE B) (Anand et al., 2018; Cosentino et al., 2020). That said, the optimal antithrombotic treatment strategy for patients with polyvascular diseases remains unresolved. In patients with asymptomatic lower extremity arterial diseases, routine antiplatelet therapy is not recommended according to the European guidelines (ESC: COR III, LOE A) (Aboyans et al., 2018). It is considered reasonable in patients having an ankle-brachial index (ABI) ≤0.90 (ACC/AHA: COR IIa, LOE C-EO) while its usefulness is uncertain in those with a borderline ABI (0.91–0.99) (ACC/AHA: COR IIb, LOE B-R) as stated in the American guidelines (Gerhard-Herman et al., 2017). In patients with asymptomatic >50% carotid artery stenosis, aspirin is the most commonly used antiplatelet therapy for the prevention of atherothrombotic complications for as long as it is well tolerated at the exception of patients at very high bleeding risk (ESC: COR IIa, LOE C) (Aboyans et al., 2018).

Vorapaxar was approved in United States in 2014 for use on top of standard antiplatelet therapy for secondary prevention in patients with a history of myocardial infarction or symptomatic PAD; however its net clinical benefit remains uncertain (ACC/AHA: COR IIb, LOE B-R) (Morrow et al., 2012; Tricoci et al., 2012; Bonaca et al., 2013; Gerhard-Herman et al., 2017). It has not gained EMA approval (Gremmel and Panzer, 2017). Its benefit must be weighed against the increase in bleeding events and it is contraindicated in patients with a history of stroke, TIA or intracranial hemorrhage. Its efficacy as a sole antiplatelet therapy is unknown and its use in the context of ACS in addition to standard antiplatelet agents showed no significant benefit (Tricoci et al., 2012).

In the light of the CSPS, CSPS2 and CASISP trials (Gotoh et al., 2000; Huang et al., 2008; Shinohara et al., 2010), cilostazol is approved in Asia, United States and Europe for the treatment of PAD patients. It improves symptoms and increases walking distance in patients with claudication in the absence of tissue necrosis or rest pain (Faxon et al., 2004; Gerhard-Herman et al., 2017; Noma and Higashi, 2018). Additionally, the arterial vasodilator effect associated with the potent, rapidly reversible antiplatelet activity of iloprost suits it for use in patients with severe PAD while preliminary clinical experience with iloprost in patients with myocardial ischemia or infarction has been disappointing. Most patients tolerate iloprost infusion rates of up to 2 ng/kg/min. It is worth mentioning that headache and flushing are common and are the suggested end-points of dose titration, as higher doses are associated with a significant incidence of gastrointestinal distress and, ultimately, hypotension.

## Ischemic Stroke and Transient Ischemic Attack

Treatment of ischemic stroke depends on its etiology and the recurrence risk (Kleindorfer et al., 2021). Intracranial large artery atherosclerosis is a common cause of stroke with a high rate of recurrence (Gorelick et al., 2008). The severity of the stenosis is also a strong predictor of recurrent stroke in the territory of the stenotic artery, with 1-year rates as high as 18% in patients with ≥70% stenosis (Kasner et al., 2006). For patients with non-cardioembolic stroke, the use of antiplatelet agents rather than oral anticoagulation is recommended to reduce the risk of recurrent stroke and other cardiovascular events (AHA/ASA: COR I, LOE A) (Powers et al., 2019). However, it should be avoided within the first 24 h following intravenous thrombolysis therapy. Antiplatelet therapy could then be initiated after exclusion of secondary hemorrhage using appropriate brain imaging (Canadian Stroke Best Practice Recommendations (CSBPR): LOE B) (Gladstone et al., 2021). In the light of CHANCE, POINT and THALES trial results (Wang et al., 2013; Johnston et al., 2018; Johnston et al., 2020), DAPT associating aspirin to clopidogrel for up to 90 days might be prescribed in patients with recent (within 30 days) stroke or TIA attributable to severe stenosis (70%–99%) of a major intracranial artery as recently published in the American guidelines (AHA/ASA: COR IIa, LOE B-NR) and the CSBPR (LOE B) (Gladstone et al., 2021; Kleindorfer et al., 2021). Triple antiplatelet therapy (aspirin + clopidogrel + dipyridamole) is harmful and should not be administered in this setting (AHA/ASA: COR III, LOE B-R) (Powers et al., 2019). In people with a non-cardioembolic minor ischemic stroke (National Institutes of Health Stroke Score (NIHSS) ≤3) or high-risk TIA (ABCD2 score ≥4) in the past 24 h, the ESO recommends 21-days of DAPT associating aspirin and clopidogrel, followed by antiplatelet monotherapy thereafter [COR strong, LOE high; recommendations developed using the GRADE standards (Guyatt et al., 2008)] (Dawson et al., 2021). In case of mild to moderate non-cardioembolic ischemic stroke (NIHSS ≤5) or high-risk TIA (ABCD2 score ≥6) in the past 24 h, 30-days of DAPT combining aspirin and ticagrelor are suggested followed by antiplatelet monotherapy thereafter (ESO: COR weak, LOE moderate) (Dawson et al., 2021).

In case of extracranial large artery as well as of aortic arch atherosclerosis, antiplatelet therapy is also recommended to reduce the risk of recurrent stroke. In patients with moyamoya disease and a history of ischemic stroke or TIA, the use of antiplatelet therapy, typically aspirin monotherapy, may also be reasonable (AHA/ASA: COR IIb, LOE C-LD) while in case of ischemic stroke caused by small vessel disease, the usefulness of antiplatelet therapy, namely cilostazol is still uncertain (AHA/ASA: COR IIb, LOE B-R) (Kleindorfer et al., 2021). In the light of the previously mentioned CSPS, CSPS2 and CASISP trials (Gotoh et al., 2000; Huang et al., 2008; Shinohara et al., 2010), cilostazol is used in Asia for secondary stroke prevention (Sahara et al., 2016) however, more randomized trials, especially in non-Asian populations, are still needed to determine its usefulness for the secondary stroke prevention. For patients with a history of ischemic stroke, AF, and CAD, the usefulness of adding antiplatelet therapy to oral anticoagulants as a secondary prevention strategy is uncertain according to AHA/ASA guidelines (COR IIb, LOE C-LD) (Powers et al., 2019) and even not recommended by the ESO (COR weak, LOE moderate) (Klijn et al., 2019). Oral anticoagulant therapy is recommended over aspirin (LOE A) and DAPT (LOE B) according to CSBPR (Gladstone et al., 2021). In patients with patent foramen ovale (PFO) with a high risk anatomic feature presenting a non-lacunar ischemic stroke of undetermined cause despite a thorough evaluation, long-term antiplatelet therapy is recommended in addition to PFO closure (AHA/ASA: COR IIa, LOE B-R; ESO: LOE A for patients aged 18–60 years, LOE B for 60–65 years and LOE C <18 or >65 years; CSBPR: LOE A) (Ahmed et al., 2019; Gladstone et al., 2021; Kleindorfer et al., 2021). In patients with ischemic stroke or TIA of unknown source with no other thrombotic history and who are found to have prothrombin 20210A or factor V Leiden mutation, elevated factor VIII levels, deficiencies of protein C, protein S, or antithrombin, or isolated antiphospholipid antibody without fulfilling the criteria of antiphospholipid syndrome, antiplatelet therapy is reasonable to reduce the risk of recurrent stroke or TIA (AHA/ASA: COR IIa, LOE C-LD) (Kleindorfer et al., 2021). When antiplatelet monotherapy is indicated, usually aspirin is used with a once daily dose. For patients with TIA, aspirin, clopidogrel, or the combination of aspirin and extended-release dipyridamole is indicated for secondary prevention of ischemic stroke (AHA/ASA: COR I, LOE A) (Kleindorfer et al., 2021). In case of high-risk TIA, DAPT (aspirin plus clopidogrel) should be initiated early (ideally within 12–24 h of symptom onset and at least within 7 days of onset) and continued for 21–90 days, followed by SAPT (AHA/ASA: COR I, LOE A; ESO COR strong, LOE high; CSBPR: LOE A) (Fonseca et al., 2021; Gladstone et al., 2021; Kleindorfer et al., 2021). Ticagrelor monotherapy among patients with high-risk TIA was also assessed in the EUCLID (Hiatt et al., 2017) and SOCRATES (Johnston et al., 2020) studies and showed no significant improvement compared to clopidogrel or aspirin, respectively. It is only recommended if aspirin is contraindicated (Powers et al., 2019) except in the Canadian guidelines in which the combination of aspirin plus ticagrelor for 30 days could be a reasonable short-term DAPT option (CSBPR: LOE B) (Gladstone et al., 2021). In case of acute non-cardioembolic low-risk TIA or of uncertain diagnosis, SAPT is recommended by the ESO (expert opinion) (Dawson et al., 2021).

The usefulness of aspirin on secondary long-term prevention in stroke and TIA patients is not well-established, nor the efficacy of clopidogrel alone, the aspirin/dipyridamole combination, ticagrelor alone, or cilostazol alone (Rothwell et al., 2016; Kleindorfer et al., 2021). Few clinical studies have also evidenced that dipyridamole alone would be effective (Diener et al., 1996; Halkes et al., 2006). That said, the CSBPR recommends antiplatelet therapy for long-term secondary stroke prevention to reduce the risk of recurrent stroke and other vascular events in patients with ischemic stroke or TIA unless there is an indication for anticoagulant therapy (LOE A) (Gladstone et al., 2021). This antiplatelet therapy consists on aspirin (80–325 mg daily), clopidogrel (75 mg daily), or combined aspirin and extended-release dipyridamole (25mg/200 mg BID) depending on patient factors and clinical circumstances (CSBPR: LOE A) (Gladstone et al., 2021).

Antiplatelet therapy in stroke and TIA patients is far from being optimized and many issues remain to be substantially addressed. The optimal time to switch from DAPT to SAPT to maximize benefit and reduce risk is not fully established. Similarly, whether other combinations than aspirin and clopidogrel are equally or more beneficial in these settings remains also uncertain. Future randomized clinical trials are thus still needed to determine the optimal combination of medications, the timing of initiation and duration of DAPT, and the effectiveness and safety of a given antiplatelet agent over another in some specific subgroups of patients according to stroke characteristics, laboratory or genetic tests or other factors.

## Primary Prevention of Cardiovascular Diseases

In light of the ASPREE, ASCEND, ARRIVE and TIPS-3 studies, efficacy and favorable benefit/risk balance of aspirin in primary prevention of CVD remains controversial (Bowman et al., 2018; Gaziano et al., 2018; McNeil et al., 2018; Yusuf et al., 2021). The United States Preventive Services Task Force (USPSTF) will no longer recommend routine prescribing of low dose aspirin for primary prevention of CVD (Mahase, 2021). It may be considered in a limited subset of United States patients aged 40–70 years at high ischemic and no increased bleeding risks (AHA/ASA: COR IIb, LOE A) (Arnett et al., 2019). In Europe, primary prevention may be considered in DM patients with high/very high risk (i.e., patients with DM having ≥1 organ damage or ≥3 major risk factors, or any risk factor and ≥10 years’ disease duration without organ damage) in the absence of clear contraindications (ESC: COR IIb, LOE A) (Cosentino et al., 2020). A recently published meta-analysis has shown that while low doses of aspirin (75–100 mg) were effective for the primary prevention of cardiovascular events in patients weighing less than 70 kg, only higher doses (300–325 mg or ≥500 mg) were so in patients weighing 70 kg or more (Rothwell et al., 2018) therefore questioning the optimal efficacy of a one-dose-fits-all approach to aspirin therapy. This meta-analysis however did not include the recent large-scale primary prevention trials ASPREE, ASCEND, ARRIVE and TIPS-3. Unlike the last three trials, ASPREE did not have the MACE as primary but rather as secondary endpoints which limits its power to assess aspirin benefit for primary MACE prevention. More recently, an individual participant data meta-analysis of 18162 participants from three large randomized controlled trials (TIPS-3, HOPE-3, and PolyIran) showed a greater reduction of MACE with the fixed-dose combination treatments (i.e., polypills associating at least two fixed-dose blood pressure lowering agents plus a statin) with vs. without aspirin at the expense of a slightly more frequent gastrointestinal bleeding events. This effect was similar at different lipid and blood pressure levels, and in the presence or absence of diabetes, smoking, or obesity (Joseph et al., 2021). Consequently, the benefit/risk balance of aspirin in primary prevention of CVD remains a topic of ongoing and future clinical trials to provide better insight regarding the ideal patient population where primary prophylactic aspirin use is beneficial. There are no trials to suggest a definite role of P2Y_12_ receptor antagonists in primary CVD prevention.

## Conclusion

Overall, antiplatelet therapy recommendations in atherothrombotic diseases are broadly similar across the guidelines. Some strategies in specific clinical settings might vary, particularly with regard to the choice of molecules, dosage, and treatment duration. This is mainly due to the availability of antiplatelet agents and their various dosages, economic considerations, public health agencies’ policies, as well as the dynamic and time-fluctuating ischemic and bleeding risks of CVD patients which highly depend on patient characteristics, including ethnicity. Moreover, many areas deserve further investigation in order to optimally manage CVD patients and provide better guidance in various medical care settings. Without any doubt, applying national and international society guidelines helps to improve CVD patient care, however, these recommendations should not be followed uniformly in the era of patient-centered precision medicine. Differences among individual patients should be considered when weighing the treatment best suited to each individual patient. With the development of novel antiplatelet agents (Jourdi et al., 2021) allegedly associated with lower bleeding risk while exhibiting at least comparable antithrombotic potency compared to the currently available drugs, antiplatelet therapy management in CVD patients is expected to change in the years ahead.
