# Supplementary material for: Antiplatelet Therapy in Atherothrombotic Diseases: Similarities and Differences Across Guidelines
Source: Front Pharmacol. 2022 Apr 27;13:878416. doi: 10.3389/fphar.2022.878416 (PMC9092185; doi:10.3389/fphar.2022.878416)
Supplement: Supplementary file 1 [file DataSheet1.docx]

**Supplementary data to**

**Antiplatelet therapy in atherothrombotic diseases: similarities and differences across guidelines**

Georges JOURDI^1,2^, Guillaume MARQUIS-GRAVEL^1,3^, Anne-Céline MARTIN^4,5^, Marie LORDKIPANIDZÉ^1,2^, Anne GODIER^4,6^, Pascale GAUSSEM^4,7^

| Quality rating | Definition |  |
| --- | --- | --- |
| High | RCT or double-upgraded observational studies | |
| Moderate | Downgraded^*^ RCT or upgraded^+^ observational studies | |
| Low | Double-downgraded^*^ RCT or observational studies | |
| Very low | Triple-downgraded^*^ RCT, downgraded^*^ observational studies or case series/case reports | |

**Supplementary Table 1: Levels of quality of a body of evidence in the GRADE approach**

RCT, randomized clinical trial

* Factors that may decrease the quality level of a body of evidence: i/- limitations in the design and implementation of available studies suggesting high likelihood of bias; ii/- indirectness of evidence (indirect population, intervention, control, outcomes); iii/- unexplained heterogeneity or inconsistency of results (including problems with subgroup analyses); iv/- imprecision of results (wide confidence intervals); or v/- high probability of publication bias.

+ Factors that may increase the quality level of a body of evidence: i/- large magnitude of effect; ii/- all plausible confounding would reduce a demonstrated effect or suggest a spurious effect when results show no effect; or iii/- dose-response gradient.

| Classes of Recommendation | | Definition |  |
| --- | --- | --- | --- |
| COR I | | Evidence and/or general agreement that a given treatment is beneficial/useful/effective | |
| COR II | **IIa** | Conflicting evidence and/or divergence of opinion about the efficacy of the treatment with a weight of evidence/opinion in favor of efficacy | |
|  | **IIb** | Conflicting evidence and/or divergence of opinion about the efficacy of the treatment with an efficacy less well established by evidence/opinion | |
| COR III | | Evidence and/or general agreement that a given treatment is not useful/effective and even potentially harmful | |

**Supplementary Table 2: Definitions of the classes of recommendation**

COR, class of recommendation

| Levels of Evidence | | Definition |  |
| --- | --- | --- | --- |
| LEO A | | Data derived from multiple RCT or meta-analyses of high quality RCT | |
| LEO B | **B-R** (randomized) | Data derived from a single high/moderate quality or multiple moderate quality RCT or from meta-analyses of moderate quality RCT | |
|  | **B-NR** (non-randomized) | Data derived from a single or multiple well-designed and well-executed non-RCT  or from meta-analyses of such studies | |
| LEO C | **C-LD** (limited data) | Randomized or non-randomized observational or registry studies with limitations of design or execution or from meta-analyses of such studies | |
|  | **C-EO** (expert opinion) | Consensus of expert opinion based on clinical experience | |

**Supplementary Table 3: Classification criteria of the levels of evidence**

LEO, level of evidence; RCT, randomized clinical trial
